# Sexual Experience, Psychological Implications, and Typical Response Strategies Among Childhood Cancer Survivors With Sexual Dysfunction in China: A Qualitative Study

**DOI:** 10.1002/pon.70396

**Published:** 2026-02-03

**Authors:** Funa Yang, Ka Yan Ho, Yaming Ji, Yan Zhai, Wenli Zuo, Xin Liu, Linlin Wang, Katherine Ka Wai Lam, Qi Liu, Ting Mao, Frankie Wai Tsoi Cheng, N. G. Chi Fai, Hongying Shi, Qi Wang, Frances‐Kam‐Yuet Wong, Janelle Yorke

**Affiliations:** ^1^ School of Nursing Hong Kong Polytechnic University Hong Kong China; ^2^ Department of Traditional Chinese and Western Medicine The Affiliated Cancer Hospital of Zhengzhou University & Henan Cancer Hospital Henan China; ^3^ Department of Hematology The Affiliated Cancer Hospital of Zhengzhou University & Henan Cancer Hospital Henan China; ^4^ Department of Orthopaedics The Affiliated Cancer Hospital of Zhengzhou University & Henan Cancer Hospital Henan China; ^5^ Department of Pediatrics and Adolescent Medicine Hong Kong Children's Hospital Hong Kong China; ^6^ Department of Surgery The Chinese University of Hong Kong Hong Kong China; ^7^ Department of Disease Prevention The Affiliated Cancer Hospital of Zhengzhou University & Henan Cancer Hospital Henan China

**Keywords:** childhood cancer survivors, psychological experience, qualitative research, response strategies, sexual dysfunction

## Abstract

**Background:**

Sexual dysfunction is a well‐documented long‐term side effect of pediatric cancer treatment, which significantly impacts the overall health of childhood cancer survivors (CCSs). There is a relative lack of qualitative research on sexual dysfunction among CCSs.

**Aim:**

This study aimed to explore sexual experience, psychological factors, and typical response strategies among CCSs with sexual dysfunction in China.

**Methods:**

A qualitative descriptive study employing semi‐structured interviews was conducted. Based on purposive sampling and data saturation principles, CCSs with sexual dysfunction from the previous cross‐sectional study were selected for semi‐structured interviews. Data relevant to the research question was analyzed and coded using thematic analysis.

**Results:**

15 female and 15 male survivors provided written‐informed consent and were interviewed in this study. Four core themes were identified, including: common sexual problems, psychological factors associated with sexual dysfunction and their related pathways, and coping strategies for sexual and psychological challenges. These themes were further categorized into 15 sub‐themes.

**Clinical implications:**

The findings of this study are expected for health professionals to develop a culturally specific intervention to improve the sexual function in childhood cancer survivors.

**Strengths & Limitations:** To our knowledge, this qualitative study is the first to provide in‐depth exploration on the sexual experience, how the identified psychological factors led to sexual dysfunction, and the coping strategies for sexual dysfunction in Chinese childhood cancer survivors. However, the findings may not be fully generalizable to older cancer survivors, and conducting interviews online may have influenced the richness of the data collected.

**Conclusions:**

The present findings contribute to our understanding of sexual experience, psychological factors, and coping strategies to sexual problems among CCSs. The findings indicate that psychology is a significant factor for CCSs with sexual dysfunction and identify the mediating role of attention and self‐compassion. Future research should develop an appropriate intervention based on the underlying psychological mechanisms of sexual dysfunction to improve the overall sexual function in CCSs.

## 1 Introduction

Sexuality is an essential physical and psychological need for humans, which has a crucial influence on quality of life, with normal sexual functioning being vital for overall well‐being [1]. Multiple factors, for example, hormone level, body image, relationships, and intimacy can affect sexual functioning, leading to the development of sexual dysfunction [2, 3]. The Diagnostic and Statistical Manual of Mental Disorders (DSM‐5) [4] provides detailed descriptions and classifications for the diagnostic criteria of sexual dysfunction. Additionally, sexual disorder is also classified as a disease condition related to sexual health in the International Classification of Diseases (ICD‐11) [5] in 2018, which includes disorders of sexual desire, arousal, orgasm, and sexual pain. As a multidimensional and complex clinical presentation, sexual issues have received an increasing attention in medical research.

Cancer represents a significant health burden for children and adolescents under the age of 18, with approximately 13.7 million new cases of childhood cancer worldwide between 2020 and 2050 [6]. Given the breakthrough in cancer diagnostic and therapeutic techniques, the population of long‐term childhood cancer survivors (CCSs) has been increasing rapidly. By 2040, the number of CCSs in the United States will reach 580,000, more long‐term survivor populations will emerge globally [7]. CCSs constitute a distinct cohort, having undergone a cancer diagnosis and treatment with chemotherapy, radiotherapy, and/or surgery during a period of accelerated physical and psychological development [8, 9]. As a result, they are particularly susceptible to the adverse effects associated with late effects of cancer treatment [8], for example, chronic diseases, social difficulties, fertility and marital problems, and self‐recognition issues, which have a detrimental impact on their quality of life and survival.

Disruption of psychosexual development is a well‐documented long‐term side effect of pediatric cancer treatment, which may contribute to sexual dysfunction. An observational study reported a delay in psychosexual development milestones [10], as well as a delay or low marriage rate [11] among CCSs, especially in female CCSs, which are closely related to sexual experiences. Previous studies [2, 12] reported a higher prevalence of sexual dysfunction among CCSs compared with the general population of similar age, with 12.30%–54.00% in male CCSs and 19.90%–57.00% in female CCSs. A large population‐based study [12] of 2546 CCSs who were young adults found that 57% of female and 35% of male survivors reported a dysfunction in at least one sexual domain measured by a self‐reported measurement. Hence, efforts are needed to optimize long‐term survivorship care for CCSs.

The etiology of sexual dysfunction is multifactorial and complex. Previous studies have attempted to identify risk factors for sexual dysfunction among CCSs, revealing associations with cancer types [13, 14, 40], gender [12, 14], mental health [15], age at diagnosis [16, 17], body image [16, 40], self‐esteem [40], and surgical history [40]. In our earlier scoping review [2], we systematically synthesized and categorized factors related to sexual function into four distinct groups, including demographic, cancer treatment‐related, psychological, and physical factors. Importantly, the results of this scoping review highlighted a significant contribution of psychological factors to sexual dysfunction among CCSs. However, no studies have yet explored the impact of psychological factors on sexual dysfunction among CCSs within the context of traditional Chinese culture. To address this research gap, we conducted a multicenter cross‐sectional study [18] to identify specific psychological factors associated with sexual dysfunction in Chinese CCSs. The results revealed that several psychological factors, including anxiety, depression, and sexual shame, were key contributing factors to sexual dysfunction, affecting both male and female survivors. These findings emphasize the potential therapeutic value of these psychological factors to tackle sexual dysfunction among Chinese CCSs.

Exploring the sexual experience of CCSs with sexual dysfunction, understanding how the identified psychological factors, including anxiety, depression, and sexual shame, contribute to sexual dysfunction, and the coping responses of CCSs with sexual dysfunction are crucial for healthcare professionals to design an appropriate intervention to improve the sexual function and sexual health of CCSs. However, most available studies that examined these issues are quantitative studies, which failed to capture the multifaceted and complex phenomenon of sexual dysfunction, especially when it does not only involve the objective presentation of sexual function, but also an individual's subjective experience, which can be influenced by culture [19]. To address this inherent limitation of quantitative studies, qualitative studies should therefore be conducted to obtain nuanced insights related to the sexual experiences of CCSs with sexual dysfunction.

To the best of our knowledge, only two qualitative studies [20, 21] have specifically addressed the topic of sexual function among CCSs. Nahata et al. [20] conducted phone interviews with 40 CCSs to examine the perceived impact of childhood cancer on their romantic relationships and sexual/physical intimacy in adulthood. The results revealed that childhood cancer experience could bring both negative and positive impacts on romantic relationships and sexual/physical intimacy. However, the impaired body image, sexual dysfunction, and reduced fertility remained significant concerns for CCSs. Another qualitative study [21] was conducted with 22 young adult survivors of childhood cancer to characterize sexual dysfunction and identify any unmet clinical need. The results provided descriptions of sexual dysfunction and identified that altered body image, reduced fertility, and inadequate clinical supports were challenges encountered by CCSs with sexual dysfunction [21]. Although these two qualitative studies offered valuable insights into this area, these studies did not address how psychological factors, that is, anxiety, depression, and sexual shame, lead to sexual dysfunction in CCSs. This is an important omission in the literature as these psychological factors are modifiable, which can be targeted by appropriate interventions, intending to improve sexual dysfunction in CCSs. Likewise, these two qualitative studies were conducted in the United States, which limited the generalizability of their findings to CCSs in other cultural contexts, for example, the Chinese population, as sexual dysfunction also involves subjective experience, which is highly dependent on culture.

In China, research on sexual dysfunction in CCSs is still relatively scarce. Different from Western countries, the Chinese society is deeply influenced by Confucianism, in which virtuous women should uphold three subordinations, which are subordinated to the father before marriage, to the husband after marriage, and to the son in widowhood [22]. Under this influence, sex is regarded as taboo in society and is prohibited from discussion. To address this specific cultural context, this qualitative study aimed to understand the sexual experience of Chinese CCSs with sexual dysfunction. Importantly, to address the gap in existing qualitative literature, we also aimed to examine how the identified psychological factors, including anxiety, depression, and sexual shame, lead to sexual dysfunction, and the coping strategies of Chinese CCSs with sexual dysfunction.

## 2 Materials and Methods

### 2.1 Research Design

A qualitative descriptive study using semi‐structured interviews was conducted. This approach is appropriate to obtain comprehensive, direct, and first‐hand descriptions of participants' experiences and perceptions of a phenomenon, especially when little is known about the phenomenon [23]. Qualitative descriptive studies stay close to the data, ensuring that participants' voices and experiences are accurately represented [24, 25]. Given these strengths, this approach was selected to achieve a comprehensive understanding of CCSs' perspectives and concerns regarding sexual dysfunction. This reporting of this study adhered to the Consolidated Criteria for Reporting Qualitative Research (COREQ) Checklist.

### 2.2 Participants

The participants were recruited by purposive sampling from our previous cross‐sectional study [18], which involved 290 CCSs who completed a survey and noted their willingness to participate in interviews. Among these CCSs, we invited those who met the eligibility criteria to join an interview via WeChat and/or phone calls. The technique of maximum variation was used to ensure the diversity of the participants in terms of age, occupation, educational level, economic status, and reproductive history.

The inclusion criteria were as follows: (a) diagnosed with cancer before the age of 18; (b) an adult who had completed cancer treatment at the time of the study; (c) no cognitive or communication difficulties; (d) a history of sexual activity with at least one sexual problem identified through assessment using the Female Sexual Function Index (a score of < 25 on the FSFI) for women [26] or the International Index of Erectile Function‐15 (a score of < 60 on the IIEF‐15) for men [27]; and e) voluntary participation in the study with a signed informed consent form. Participants were excluded if they had serious comorbidities (e.g., heart, brain, liver, or kidney diseases) or psychological disorders. The sample size was determined based on data saturation, which is defined as a point at which no new information or themes emerged during ongoing data collection [28, 29]. Ultimately, 30 participants were selected and completed the interviews.

### 2.3 Data Collection

A research assistant contacted the participants to schedule the time and method of the interviews. Given the sensitivity of the study topic, that is, sexual dysfunction, the participants were only given an option to undergo the interview either using an online platform or via phone calls. In the end, all the participants opted to have the interviews online, utilizing either the WeChat video calls or Tencent conferences.

Data was collected by the semi‐structured interviews according to the semi‐structured interview guide (Table 1) designed by the research team based on the generic conceptual framework of chronic disease and sexuality by Verschuren [30], supplemented by findings from our scoping review and cross‐sectional study in CCSs. To ensure that the participants could freely express their sexual issues, we had two interviewers, that is, one was male (YMJ), and another was female (FNY), to match the participants' gender. The two interviewers were knowledgeable in cancer survivorship care, had experience in taking care of CCSs, and were trained by the research team in qualitative research, including conducting semi‐structured interviews.

**TABLE 1:** The semi‐structured interview outline.

Introduction: How do you feel about the diagnosis and treatment of cancer in childhood?
Formal interview: 1. May I ask how your sexuality has been recently? What sexual problems have you encountered? 2. Could you share your attitudes and perceptions regarding sexuality, please? 3. Could you share your psychological feelings about sexuality? 4. What factors do you think might influence your sexual function? Why? 5. What coping strategies do you use when faced with psychological problems or sexual issues?

Given the sensitivity of sex‐related topics, the interviews began with some general questions. For example, the participants were asked to describe their feelings and experiences in relation to their cancer diagnosis and treatment. After establishing the rapport, the interviewer raised more specific questions which could be categorized into four different areas: (1) experience of recent sexual activities and associated issues and concerns; (2) psychological factors potentially leading to sexual dysfunction; (3) mechanisms and pathways that explained the impact of psychological factors on sexual functioning; and (4) their coping strategies to sexual dysfunction and sex‐related issues. Each interview was audio‐recorded with the participant's consent. Throughout the interviews, field notes were taken. The qualitative descriptions were combined with observations of actions, expressions, and other nonverbal cues, ensuring a comprehensive and nuanced understanding of the participants' experiences. All interviews lasted between 20 and 39 min, were audio‐recorded with the participant's consent, and subsequently transcribed verbatim.

### 2.4 Data Analysis

Interview recordings were transcribed verbatim within 24 h after each interview. Any discrepancy in the transcripts was resolved by repeatedly listening to the recordings and verifying the exact wording with the interviewers. Once the accuracy of the transcripts was confirmed, the data was analyzed using a thematic analysis approach [31]. The first author developed an initial codebook through repeated reading and coding of the interview transcripts. Similar codes were grouped into categories and then organized into broader themes. The team responsible for coding included the first author, one PhD candidate in nursing (TM), one postdoctoral fellow (QL), one assistant professor (KKWL), and one associate professor (KYH), all of whom have experience in cancer care and qualitative research. They reviewed and revised the codebook during weekly meetings until a consensus was reached. After multiple rounds of discussion and refinement, the first author translated the transcripts, codes, categories, and themes into English. NVivo (14, QSR) assisted in promoting the encoding process.

## 3 Rigors

To uphold the rigors of this study, we adhered to the criteria outlined by Guba and Lincoln [32] to ensure credibility, transferability, confirmability, and dependability. Credibility was addressed through multiple strategies. First, to establish rapport and elicit rich data, interviewers employed empathetic techniques (e.g., maintaining a non‐judgmental stance, observing non‐verbal cues). Second, to ensure analytical rigor, two researchers with extensive experience in qualitative research and oncology care independently coded the interview transcripts. The coding team then met weekly to compare codes, discuss discrepancies, refine the codebook, and reach consensus on theme development, a process of researcher triangulation. Third, verbatim transcription was completed within 24 h to ensure accuracy. Finally, to address translation concerns, a robust translation and back‐translation process was employed. Transferability was ensured by employing purposive sampling to maximize participant diversity (e.g., in age, gender, cancer type). Detailed descriptions of the context, procedures, and participant characteristics are provided to allow readers to assess the applicability of our findings to other settings.

Dependability and Confirmability were strengthened through triangulation and reflexivity. The research team engaged in continuous self‐reflection and held regular consultations to discuss interpretations and minimize bias. Furthermore, member checking was conducted: two participants were invited to review and provide feedback on the preliminary thematic structure, which confirmed that our findings accurately reflected their experiences. This process, along with the detailed audit trail provided in our methods, enhances the confirmability of the study.

## 4 Ethics

This study was conducted following the ethical standards outlined in the Helsinki Declaration and received approval from the Hong Kong Polytechnic University (HSEARS20240918003). All participants were informed that their participation was entirely voluntary and that their personal information would remain strictly confidential, accessible only to the research team. Written informed consent was obtained from each participant before the interview. As a token of appreciation, 100 RMB was provided to each participant upon completion of the interview.

## 5 Results

A total of 55 eligible participants were invited for the interviews, of whom 17 (30.9%) declined. Among the remaining 38 individuals who expressed interest, 30 were ultimately interviewed; the others were unable to participate due to scheduling conflicts. The participants comprised 15 females and 15 males, with a mean age of 28.3 years (ranging from 20 to 43). The majority identified as heterosexual, except one homosexual and one bisexual. Sixteen participants (53.3%) were diagnosed with leukemia, while others had lymphoma (5/30, 16.7%), bone tumors (5/30, 16.7%), brain tumors (2/30, 6.67%), osteosarcoma (1/30, 3.33%), and uterine tumors (1/30, 3.33%). Detailed demographic and disease characteristics of the participants are presented in Table 2.

**TABLE 2:** The demographic and clinical characteristics of informants.

Informants	Demographic characteristics								Clinical characteristics					
	Age	Religious	Residence	Education	Relationship status	Reproductive	Occupational	Sexual orientation	Age at diagnosis	Diagnosis	Surgery	SCT	Radiotherapy	Menstruation
F1	25	Yes	Cities	College	Cohabitation	None	Unemployed	Heterosexual	16	Leukemia	No	Yes	Yes	Yes, normal
F2	27	Yes	Cities	High school	Married	Two children	Unemployed	Heterosexual	16	Osteosarcoma	Yes	No	Yes	Yes, not normal
F3	28	No	Villages	College	Single	None	Employed	Heterosexual	15	Leukemia	No	Yes	Yes	No
F4	38	No	Cities	Middle school	Single	None	Unemployed	Heterosexual	16	Leukemia	No	Yes	Yes	No
F5	23	No	Villages	College	Single	None	Unemployed	Heterosexual	12	Lymphoma	Yes	Yes	Yes	No
F6	22	No	Cities	College	Single	None	Unemployed	Homosexual	16	Bone tumors	Yes	No	No	Yes, normal
F7	23	No	Villages	Middle school	Single	None	Employed	Heterosexual	15	Bone tumors	Yes	No	No	Yes, not normal
F8	27	No	Cities	College	Single	None	Employed	Heterosexual	12	Lymphoma	No	No	Yes	No
F9	21	No	Cities	College	Single	None	Sick leave	Heterosexual	15	Leukemia	No	Yes	Yes	No
F10	27	No	Villages	High school	Married	One child	Employed	Heterosexual	16	Brain tumor	Yes	No	Yes	Yes, normal
F11	40	No	Villages	Middle school	Married	One child	Unemployed	Heterosexual	15	Leukemia	Yes	Yes	Yes	No
F12	43	No	Cities	Middle school	Married	One child	Unemployed	Heterosexual	16	Uterine tumor	Yes	No	Yes	No
F13	28	No	Cities	College	Married	One child	Unemployed	Heterosexual	15	Leukemia	Yes	Yes	Yes	No
F14	36	No	Villages	Middle school	Married	None	Sick leave	Heterosexual	16	Leukemia	No	Yes	Yes	Yes, not normal
F15	35	No	Villages	College	Cohabitation	None	Unemployed	Heterosexual	16	Leukemia	Yes	Yes	Yes	Yes, not normal
M1	23	No	Villages	College	Single	None	Employed	Bisexual	8	Bone tumor	Yes	No	No	—
M2	29	Yes	Cities	High school	Cohabitation	None	Employed	Heterosexual	14	Bone tumor	Yes	No	Yes	—
M3	24	No	Cities	College	Single	None	Employed	Heterosexual	15	Leukemia	No	No	Yes	—
M4	22	No	Cities	High school	Single	None	Unemployed	Heterosexual	15	Leukemia	Yes	Yes	Yes	—
M5	23	No	Cities	College	Single	None	Unemployed	Heterosexual	10	Leukemia	No	No	Yes	—
M6	35	No	Villages	High school	Married	One child	Sick leave	Heterosexual	14	Leukemia	No	Yes	Yes	—
M7	27	No	Villages	College	Single	None	Employed	Heterosexual	14	Lymphoma	No	No	Yes	—
M8	35	No	Cities	University	Married	One child	Employed	Heterosexual	16	Lymphoma	Yes	No	Yes	—
M9	25	No	Villages	College	Single	None	Employed	Heterosexual	16	Bone tumors	Yes	No	No	—
M10	20	Yes	Cities	High school	Single	None	Unemployed	Heterosexual	15	Leukemia	No	Yes	Yes	—
M11	20	No	Cities	College	Single	None	Unemployed	Heterosexual	15	Lymphoma	Yes	No	Yes	—
M12	34	No	Villages	High school	Married	One child	Unemployed	Heterosexual	15	Leukemia	Yes	Yes	Yes	—
M13	23	No	Villages	College	Single	None	Unemployed	Heterosexual	13	Leukemia	No	Yes	Yes	—
M14	25	No	Towns	High school	Single	None	Unemployed	Heterosexual	14	Leukemia	No	No	Yes	—
M15	42	No	Villages	University	Married	One child	Employed	Heterosexual	15	Brain tumor	Yes	No	No	—

Through sorting, analyzing, and coding of the interview data, four core themes were identified. They were common sexual problems, common psychological factors that lead to sexual dysfunction, pathways explaining the impact of psychological factors on sexual functioning and coping strategies for sexual and psychological challenges. There were also 15 sub‐themes under these four core themes, as detailed in Table 3.

**TABLE 3:** The main themes of qualitative interview.

Theme	Subtheme
Theme 1: Common sexual problems	Lack of sexual interest
	Poor sexual satisfaction
	Inability to achieve orgasm
	Insufficient erections in terms of duration, frequency and intensity
Theme 2: Psychological factors contributing to sexual dysfunction	Anxiety and depression
	Sexual shame
	Body image disturbance
	Low self‐esteem
Theme 3: Pathways of psychological factors leading to sexual dysfunction	Impaired attention during sexual activities
	Lack of self‐compassion
	Complex interplay between physiological and psychological factors
Theme 4: Coping strategies for sexual dysfunction	Enhancing intimacy
	Self‐emotional adjustment
	Increasing awareness of self‐inadequacy
	Self‐value clarification

### 5.1 Theme 1: Common Sexual Problems

Most participants reported a range of sexual problems, with these problems interacting with each other and varying by gender. Among the female participants, the three most commonly reported problems were loss of sexual interest, inability to achieve orgasm, and lack of sexual satisfaction. While for male participants, the three most commonly reported problems were lack of sexual interest, insufficient erections in terms of duration, frequency, and intensity, and poor sexual satisfaction.

#### 5.1.1 Subtheme 1.1: Lack of Sexual Interest

Both female and male participants reported a decline in sexual interest, though this issue appeared to be more pronounced in our female participants. Many female participants described themselves as frigid, both physically and psychologically. They expressed that sex was dispensable, and sexual interest could sometimes lead to the feelings of disgust and repulsion. In the semi‐structured interview, one female participant said,

I am kind of frigid in sex matters and don't feel very comfortable with it. Sex, for me, is just dispensable. When I am alone, I will not think about that, and when I am with my partner, I am not the one who mentions it first.

(F5)

Another female participant who experienced a decrease in sexual interest stated that,

Since I was 40, I haven’t had any sex life. Basically, not for half a year. It seems that sexual needs decline with age, even becoming a feeling of pain, which makes me resist it.

(F12)

Similarly, some male participants also indicated a lack of interest in sexual matters. Despite most did not express something negative toward sex, they did not present a positive attitude either. One male participant shared his thoughts about sexual interest,

I am not that positive toward this stuff (sex), and not an active person about sex things. It seems that it (sex) is not important in daily life. I could go for 2–3 months without those activities (sex) when I was not able to do it physically.

(M6)

#### 5.1.2 Subtheme 1.2: Poor Sexual Satisfaction

Both female and male participants reported low sexual satisfaction, and such problem appeared to be more significant in female participants. During the interviews, female participants frequently described experiencing significant dissatisfaction with their sexual lives, using terms like “struggling” or “not enjoyment” to characterize their experiences. Male participants also reported dissatisfaction, with several describing their sexual experiences as “not that good”.

One female participant said this in the semi‐structured interview,

It feels like I am struggling to finish a task (when having sex). I don’t have that much feeling during sex, I mean, I don’t have enjoyment and happiness, which should be related to my poor physical condition.

(F13)

Another male participant expressed similar concerns,

Sex is not as wonderful as I thought before, it has become not that good and just adequate in life.

(M7)

#### 5.1.3 Subtheme 1.3: Inability to Achieve Orgasm

This problem was exclusively mentioned by some female participants. They mentioned that they seldom or even never achieved orgasm. Some also mentioned that they were uncertain about the physical sensation of orgasm, and hence they did not know whether they achieved it or not. The female participants who reported the difficulties in achieving orgasm attributed this problem to their diminished sexual interest and the presence of discomfort during sexual activity. One female participant said,

I don't know whether I can reach orgasm. It seems that I do not have such feelings since I was young because I'm not really interested in sex.

(F2)

Another female participant similarly said,

I am confused (with the weak orgasm). I could not bear to have sexual activities for a long time as it makes me uncomfortable and painful. But I also doubt, maybe it (the sexual activity) is not long enough to achieve orgasm.

(F6)

#### 5.1.4 Subtheme 1.4: Insufficient Erections in Terms of Duration, Frequency, and Intensity

Most male participants reported concerns regarding erectile dysfunction, characterized by diminished erectile strength, shortened duration, and/or reduced frequency. These issues seemed to be more prominent in male informants who were older. Additionally, some male participants highlighted that emotional symptoms and declining passion might contribute to this problem.

One male participant said,

The main concern is the hardness. I feel it isn't hard enough. I mentally resist sex life when it occurs in my head. As long as I think that I may not be able to get hard, the picture comes into my mind, and I keep repeating it. I am anxious every time before sex.

(M14)

Another male participant also said,

It’s just not enough in intensity and frequency (for erection). When I had just got married, I had more sex due to the passion. And now, almost like 1‐2 times a month.

(M6)

### 5.2 Theme 2: Psychological Factors Contributing to Sexual Dysfunction

In the interviews, a range of psychological issues have been identified, which could contribute to sexual dysfunction among CCSs. The psychological issues included anxiety and depression, body image disturbance, sexual shame, and low self‐esteem. Also, these psychological issues were found to be more pronounced in female participants than male participants.

#### 5.2.1 Subtheme 2.1: Anxiety and Depression

The interview revealed a high prevalence of anxiety and depression among both female and male participants, which significantly impaired their sexual functioning and performance. The primary sources of anxiety and depression encompassed physical discomfort associated with sex and concerns related to marriage and fertility. In the interviews, some female participants mentioned that anxiety and depression led them to be very passive in sex, affecting their performance and satisfaction. For male participants, some indicated that anxiety and depression made them feel stressed, which then contributed to erectile dysfunction, leading them to reduce sexual frequency, ultimately precipitating distress to their partners.

One female participant elaborated on this issue in the semi‐structured interview,

I always worry about not performing well during sex. This anxiety makes me feel tense ‐ both physically and mentally ‐ and it's really hard for me to relax in that moment (sex).

(F9)

One female also experienced similar concerns,

I feel I lose interest in everything, probably depression. Sex? I only do it because I feel like I have to.

(F5)

Another male participant also said,

It (anxiety) definitely affects my sexual performance in a certain extent. There were several occasions when… well, the anxiety just made me fail to erection.

(M3)

#### 5.2.2 Subtheme 2.2: Sexual Shame

Most of the female and male participants reported sexual shame. They perceived sexual activity as morally inappropriate and felt embarrassed when discussing it openly. Some participants also stated that even if they have experienced sexual dysfunction that required assistance, they would not disclose such issues due to the personal and private nature of the topic. The participants said their reluctance in discussion might be due to their negative parental attitudes toward sex, family values, and societal education regarding sex. As one female participant stated:

I feel sex, or talking about sex in public, is embarrassing. Sex seems like a bad thing. When I have sex, it feels like I am doing something bad on the sly.

(F7)

Another male participant further illustrated,

If I have any issue with sex, I’d probably just leave it …Honestly, the idea of going to the hospital to see a doctor for something like that just makes me uncomfortable.

(M10)

#### 5.2.3 Subtheme 2.3: Body Image Disturbance

Body image disturbance was predominantly reported by female participants, who expressed dissatisfaction with their physical appearance due to surgical scars, breast development, walking posture, facial aging, and excessive thinness. Only one male participant mentioned concerns about his unattractive appearance. Such dissatisfaction with body image led to diminished self‐confidence, which directly or indirectly impacted their sexual function. One female participant said,

My physical attractiveness is relatively low due to my small boobs, which seem not sexually mature. Another thing is that I get necrosis of the femoral head and hence my legs are in unbalanced strength. I also get scoliosis. All these make me look crooked. When I go out, I feel like people are looking at me, and I have some feelings of inferiority…

(F7)

Another female participant also said,

I always feel that I am not beautiful. I just look a bit older than my peers, and other people would reject me for being too skinny. Anyway, there is nothing about my body that I am satisfied with (Laughs bitterly).

(F11)

#### 5.2.4 Subtheme 2.4: Low Self‐Esteem

Low self‐esteem was prevalent in both female and male participants, though its manifestations differed by gender. Male participants with low self‐esteem often projected a lack of self‐confidence and perceived themselves as failure, which caused them to be reluctant to have sex.

As articulated by a male participant:

Ever since that one time I didn’t perform well, I’ve been really resistant to it (sex)… I mean, if I can’t even get things right in life, how could sex be any different?

(M10)

While female participants with low self‐esteem also focused on their self‐image, and often conceived a negative self‐image from their partners, which consequently compromised their attractiveness, sexual performance, and satisfaction. In the semi‐structured interview, a female participant said,

When we have sex every time, I wonder if he's judging my body… These thoughts make me impossible to relax, let alone enjoy the moment (sex).

(F12)

### 5.3 Theme 3: Pathways of Psychological Factors Leading to Sexual Dysfunction

Three pathways were identified that could explain how the identified psychological factors led to sexual dysfunction. The three pathways included (1) impaired attention during sexual activities, (2) lack of self‐compassion, and (3) complex interplay between physiological and psychological factors.

#### 5.3.1 Subtheme 3.1: Impaired Attention During Sexual Activities

A majority of participants indicated that their anxiety and depression, sexual shame, and body image disturbance significantly impaired their attention when they had sexual activity. Particularly, they were kept distracted by these emotions and thoughts and were unable to relax. Hence, they could not concentrate on sex, enjoy sex, achieve orgasm, and eventually reduce sexual satisfaction.

One participant explained this situation,

During sex, I always think about my past unpleasant experiences, and worry whether my body will have problems. I can’t stop thinking about it. It’s really hard for me to enjoy the moment.

(F14)

Another participant also shared the emotions and thoughts when having sex,

I can't help associating it (sex) with dirty… These thoughts make my mind wandering. It's really hard for me to focus on it (sex).

(M15)

One more participant further illustrated how she was distracted during sex,

Because of my poor body image, I am always worried about my performance in sex and whether I can satisfy my partner. The worry and anxiety make me hard to relax in sex.

(F3)

#### 5.3.2 Subtheme 3.2: Lack of Self‐Compassion

Despite the participants had experienced a lot of psychological challenges in having sex, some did not demonstrate self‐compassion was characterized as insufficient caring about themselves and prioritizing their partner's sexual needs over their discomfort and reluctance in sex. Also, as stated in the interviews, they heavily emphasized the responses of their partners. If their partners showed any sign of dissatisfaction, they would be anxious and think they had done something wrong in the process.

A female participant in the semi‐structured interview highlighted this,

I am not good enough…When there is something wrong during sex, I will stay quiet because I don't want to kill the mood of my partner.

(F10)

Another participant also said,

Honestly, if I can't even stand my own body, I also understand why my partner cannot accept my body. I am unable to fulfill my role as a spouse. So, this is normal for him to blame me.

(M4)

#### 5.3.3 Subtheme 3.3: Complex Interplay Between Physiological and Psychological Factors

Participants consistently identified some physiological factors, particularly ovarian insufficiency and chronic fatigue, contributing significantly to sexual dysfunction. Likewise, these physiological factors interacted with the participants' negative emotions, anxiety, depression, stress, and low self‐esteem to affect their sexual functioning. Particularly, the negative emotions affected the secretion of sex hormones, leading to sexual dysfunction, such as vaginal dryness and insufficient blood flow for penis erection, exacerbating discomfort and difficulties during sexual activity.

As articulated by one participant in the semi‐structured interview,

I am upset and irritable all day, I feel like I am in the period of ‘premature ovarian failure’, I have experienced a lot of symptoms which look like menopause. All these issues add together to affect my hormone levels. My vagina is dry and appears to be shrunken.

(F13)

Similar descriptions have been reiterated by another participant,

The doctor said my hormone levels are even lower than those of women in menopause. This affects me in so many ways, including my emotions, which probably causes me to have no sexual desire.

(F6)

### 5.4 Theme 4: Coping Strategies for Sexual Dysfunction

The participants reported various coping strategies to address sexual dysfunction and associated psychological distress. These coping strategies included enhancing intimacy, self‐emotional adjustment, increasing awareness of self‐inadequacy, and self‐value clarification.

#### 5.4.1 Subtheme 4.1: Enhancing Intimacy

To overcome sexual dysfunction, both male and female participants emphasized enhanced intimacy as a crucial component. Particularly, they thought that spousal communication and collaborative coping were important to convey their own expectations, difficulties, and pleasurable ways regarding sexual activities. These could help them overcome the challenges associated with sexual dysfunction (e.g., vaginal dryness, lack of sexual interest, and failure to achieve orgasm) together, thus improving sexual satisfaction.

This was emphasized by one male participant in the semi‐structured interview,

Encouragement, understanding, cooperation, and expressing love with each other are the foundation of sex. It would be pleasurable and joyful to have sex only under those conditions.

(M4)

One female participant also expressed how enhanced intimacy helped her cope with sexual dysfunction,

Once my husband stopped treating sex as a chore—when he actually listened to my worries and helped me deal with the dryness—I finally felt close to him instead of just pressured to have sex.

(F7)

#### 5.4.2 Subtheme 4.2: Self‐Emotional Adjustment

Some participants mentioned that they have adopted various strategies to manage their negative emotions, for example, anxiety and disrupted body images that might contribute to their sexual dysfunction. Particularly, some participants mentioned that they have strived to have sexual activities that are the same as those of other normal people, and this resulted in a lot of anxiety and stress for them. Finally, they accepted the fact that they did have some impairment in sexual functioning due to childhood cancer, which could not be recovered, and became more Buddha‐like, lowering my expectations. Consequently, when they accepted this, they felt less anxious and even found sexual activities to be more enjoyable.

This was emphasised by one participant in the semi‐structured interview as follows,

After years of forcing myself to meet the 'normal' standards, I finally realized that it (sex) cannot be forced. I only have to focus on the good parts, rather than the bad parts.

(F9)

Another participant similarly said,

In fact, cancer is not a problem for me now. I cannot control how it (sex) will be. The best I can do is be positive towards it.

(M8)

One other participant also said,

If there was good sex in my life, that would be great. If not, I also would not force myself.

(F5)

#### 5.4.3 Subtheme 4.3: Increasing Awareness of Self‐Inadequacy

Some participants mentioned that they have encountered a lot of emotions and thoughts that were not favourable for sexual activities. To cope with this issue, they have started to pay more awareness on these emotions and thoughts, particularly when having sexual activities. They thought that the increased awareness of these emotions and thoughts could facilitate them to interpret and control their own bodily sensation, eventually improving their sexual responses and interest.

As illustrated by one participant in the semi‐structured interview,

I’ve started paying more attention to my own feelings during sex. There may be pain in my genital area sometimes. I see it as part of my bodily sensations. I now can feel, recognise, adapt to it. This makes me more enjoy my life.

(F6)

One informant accepted his negative emotions and stated that,

When I stopped fighting with the anxiety, this feeling fade, without any reason. I finally got it, the performance of sex should not be graded or evaluated.

(M2)

#### 5.4.4 Subtheme 4.4: Self‐Value Clarification

Some participants reported that they felt less anxious and depressed after re‐evaluating their life goals. Particularly, they found that their self‐worth should not be defined by sexual life and sexual function. Conversely, they thought that they should look beyond sex and find meanings in other domains of life.

As illustrated by one informant in the semi‐structured interview,

Personally, I often reflect on what the meaning of life is. My value as a person isn't defined by my sexual life.

(M8)

Another informant similarly said,

It took me a while to realize ‐ the good stuff in my life isn't about (sex) perfection. Just being mindful, soaking up the moment, having real connections with family… that's what actually matters.

(F9)

## 6 Discussion

As CCSs enter into adulthood, they begin to form intimate relationships and engage in sexual activity. However, existing evidence has indicated that their sexual function is often impaired to different degrees due to several psychological factors, including anxiety, depression, sexual shame, disrupted body image, and low self‐esteem [2]. Likewise, when compared to Western countries, the public attitudes toward sex remain more conservative in China, due to the influence of Confucianism. To the best of our knowledge, this qualitative study is the first to provide an in‐depth exploration of the sexual experience, how the identified psychological factors led to sexual dysfunction, and the coping strategies for sexual dysfunction in Chinese childhood cancer survivors. The findings are expected to guide the development of a culturally specific intervention to improve sexual function in this population.

In this qualitative study, both female and male participants reported reduced sexual interest and low sexual satisfaction as their common sexual problem. This finding was in line with a previous cross‐sectional study [33], which documented that 30% of CCSs experienced diminished sexual desire, while 24% struggled with sexual enjoyment. Although the female and male participants did share some common sexual problems, there were problems specific to gender. As indicated in our semi‐structured interviews, female participants more frequently reported the inability to achieve orgasm, while male participants reported insufficient erections in terms of duration, frequency, and intensity. The gender differences could be explained by the anatomical and functional differences in the sex organs. One thing worth noting is that most participants reported more than one sexual problem, and these sexual problems were mostly interrelated and interacted with each other. For example, some female participants who complained of sexual discomfort also reported that such discomfort reduced their sexual interest and hindered their ability to achieve orgasm. This underscores the complexity of sexual dysfunction in CCSs in which symptoms may cascade and amplify one another. Consequently, identifying the central symptom, which is defined as the most important symptom that cascades and amplifies other co‐occurring symptoms, could inform more precise and effective interventions for sexual dysfunction.

Our qualitative study identified that some physical adverse effects associated with cancer and its treatment were highly associated with sexual functioning in CCSs. The two most frequently cited adverse effects of the participants were premature ovarian failure and fatigue. According to existing data, the prevalence of premature ovarian failure is only 1% in the general population, whereas that in female CCSs is nearly 10% [34], which can directly disrupt hormonal balance and impact sexual and reproductive functions [35]. Another commonly reported late effect was chronic fatigue, which can cause neuroendocrine disruption and hormonal imbalance, further damaging sexual function. Importantly, as revealed by our study, these adverse physical effects led to various psychological responses among CCSs, collectively affecting their sexual function. These findings concur with our previous cross‐sectional study in this population, which showed that psychological responses did play a significant role in contributing to sexual dysfunction, despite it is a problem resulting from multiple factors.

Of all psychological responses, our study found that anxiety and depression were important contributors to sexual dysfunction in CCSs, and these psychological responses partly originated from the uncertainty regarding their marriage and fertility. As noted in our previous scoping review, CCSs always report a delay in their developmental milestones, including their marriage and sexual relationships [2]. Additionally, some evidence showed that approximately one‐third of the CCSs experience infertility [36], which led many CCSs to become uncertain about their fertility until they give birth to a child [37]. One thing that warrants our attention is that the relationship between these psychological responses and sexual dysfunction is not causal, but rather bi‐directional, in which CCSs with sexual dysfunction exhibit heightened anxiety and depression, leading to a vicious cycle to further exacerbate sexual dysfunction [21]. To break this vicious cycle, it is therefore crucial to develop and implement appropriate interventions to target these negative psychological responses among CCSs with sexual dysfunction, to improve their sexual function and quality of life.

Apart from anxiety and depression, sexual shame was also reported by our participants as a contributing factor to sexual dysfunction, which concurs with the results of our previous cross‐sectional study [38]. Our qualitative findings further illustrate the participants' conservative beliefs in relation to sex and sexual dysfunction under the influence of Confucianism. Importantly, due to their beliefs, they were reluctant to discuss and disclose sexual dysfunction to healthcare professionals and, therefore, were unable to receive appropriate and timely interventions. Likewise, attributing to cancer and its treatment, quite a number of participants mentioned low self‐image due to their affected appearance, which in turn reduced their self‐confidence to have sex with their partners. However, unlike previous studies, which found that sexual dysfunction caused low self‐confidence [39], our semi‐structured interview revealed that low self‐esteem could lead to sexual dysfunction in CCSs, rather than a result of sexual dysfunction. This finding brings us a new insight to re‐evaluate the relationship between self‐esteem and sexual function. In fact, a considerable number of studies have pointed out that low self‐esteem could be a result of sexual dysfunction, as sexual function is an indicator of personal attractiveness [40]. However, from our qualitative findings, the relationship between self‐esteem and sexual function should be bi‐directional rather than causal, in which low self‐esteem in sexual activities could also reduce individuals' interest in sex and impair their performance in the process, resulting in sexual dysfunction. Given the conservative beliefs about sex in Chinese society, the psychological interventions should be culturally specific to this population group. In addition, how to improve self‐esteem in sex and body image should also be taken into account in the interventions.

In our qualitative study, we also explored how the negative psychological responses translated into an impact on the sexual function of CCSs. One possible explanation was impaired attention in sexual activities due to these negative psychological responses. In the semi‐structured interviews, quite number of participants reported that they have experienced various negative emotions and flashbacks to bad sex experiences when having sex with their partners. These emotions and memories made their mind wander and put them under stress throughout the process, which in turn reduced their awareness of sexual stimulations and impaired their performance, interest, and satisfaction in sex. This finding concurs with the cognitive‐affective model of sexual dysfunction proposed by Barlow (1986), which indicates that optimal sexual functioning builds on the foundation of adequate attention to facilitate the awareness of erotic stimuli, cognitive processing of sexual cues, and emotional engagement. When attention is compromised, the cascade of psychosexual processes will be disrupted, resulting in sexual dysfunction [41].

To cope with the psychological challenges, multiple strategies, including emotional adjustment, self‐value clarification, and increasing awareness of self‐inadequacy, have been reported by the participants. Coincidentally, when compared with existing literature, these three coping strategies correspond to the three core components of mindfulness, including intention, attitudes, and attention [42]. For intention, it emphasizes cultivating the awareness [42], which echoes the CCSs who paid more awareness on their emotions and thoughts that could lead to sexual dysfunction. For attitude, it focuses on non‐judgmental and being kind [42], which are corresponding to the Buddha‐like attitude adopted by CCSs with sexual dysfunction. Concerning the attention, it highlights the present moment [42], which is also in line with the CCSs who encouraged themselves to enjoy life despite sexual dysfunction. These findings suggest that mindfulness‐based interventions may be potentially effective in mitigating the psychological factors contributing to sexual dysfunction among CCSs, as this intervention approach exactly addresses their coping characteristics. In fact, mindfulness‐based interventions have been applied to improve sexual function in cancer survivors, with positive findings noted in systematic reviews and randomized controlled trials [43, 44, 45]. However, due to the distinctive differences in CCSs from other populations, especially the disrupted puberty and psychosexual development due to the treatment of cancer, it is crucial to examine the effectiveness and feasibility of mindfulness‐based interventions in promoting sexual dysfunction and alleviating its associated psychological distress in CCSs.

This study also revealed that a lack of self‐compassion among CCSs could be another possible explanation that translated the psychological distress, for example, low self‐esteem, into actual impacts on their sexual function. According to Neff (2003), self‐compassion refers to supporting oneself when experiencing pain and suffering caused by personal mistakes and shortcomings. An increasing number of studies have shown that self‐compassion can reduce the symptomatology associated with anxiety, depression, and psychological distress [46, 47, 48] through increasing the self‐kindness to personal mistakes and shortcomings and becoming less judgmental of their own faults. Some evidence also suggested that self‐compassion could mediate the psychological impact of childhood trauma by assisting individuals to overcome unpleasant thoughts, improving their own acceptance and perception of body image, eventually enabling them to build up a more intimate relationship with their partners [49]. Despite the importance of self‐compassion, as indicated in the semi‐structured interviews, a majority of participants felt ashamed to have sexual dysfunction, denied their own values because of failure to satisfy the partners' sexual needs, and, importantly, prioritized their partners' needs and emotions over their needs and emotions. Hence, apart from mindfulness‐based interventions, cultivating self‐compassion among CCSs is equally important to mitigate the impact of psychological distress on their sexual dysfunction.

A noteworthy finding was that most participants had tried to develop an intimate partner relationship to cope with negative emotions associated with sexual dysfunction. This observation aligns with the theory of dyadic illness management, which posits the patient and the informal care partner, usually the spouse, as a dyad to appraise the symptoms. Based on the dyadic appraisal, they will jointly manage the symptoms, eventually affecting both the patient's and the informal care partner's health [50, 51]. Since sex is not something that can be achieved by individuals, involving the spouses of CCSs with sexual dysfunction in the interventions is important to improve the feasibility and effectiveness.

### 6.1 Clinical Implications

Our study has noteworthy strengths for clinical practice. First, the study has illuminated the lived insights of CCSs regarding their sexual experience. Second, the study findings corroborate existing quantitative evidence, which mainly focuses on prevalence and risk factors of sexual dysfunction of CCSs, adding to our understanding of how negative psychological responses led to sexual dysfunction, and their corresponding coping strategies. Therefore, this qualitative evidence is vital to inform healthcare professionals on the development of psychological interventions to manage sexual dysfunction among CCSs.

## 7 Limitations

Despite these unique findings, this study had some limitations. Firstly, although we aimed to diversify the sample in terms of their demographic characteristics, the participants were predominantly young individuals. Only three participants were aged more than 40 years. This compromised the generalizability of the study results to CCSs at older ages. Secondly, all the interviews were conducted online, which may have reduced the richness of data collection, especially the non‐verbal cues.

## 8 Conclusion

The present findings contribute to our understanding of sexual experience, psychological responses contributing to sexual dysfunction, and coping strategies among CCSs with sexual dysfunction through in‐depth interviews within the context of Chinese culture. Negative psychological responses, such as anxiety, depression, disrupted body image, and sexual shame, were found to reduce attention of CCSs when they had sex, alongside low self‐compassion among them to their own pain and suffering, causing sexual dysfunction. Based on the coping strategies used by CCSs, mindfulness‐based interventions are a potential treatment option to enhance their sexual functions. Due to the distinct culture and characteristics of CCSs, rigorous studies are warranted to confirm the feasibility and effectiveness of this intervention approach before a large‐scale implementation.

## Author Contributions

Conceptualization: F.N.Y., K.Y.H., Y.Z., W.L.Z., X.L., L.L.W., F.W.T.C., N.C.F. Methodology: F.N.Y., K.Y.H. Investigation: F.N.Y., Y.M.J. Data curation: F.N.Y., H.Y.S. Formal analysis: F.N.Y., K.Y.H., K.K.W.L., Q.L., T.M. Writing–original draft: F.N.Y. Writing–review and editing: K.Y.H, Q.L., Q.W., J.Y. Supervision: K.Y.H., F.K.Y.W., J.Y. Project administration: F.N.Y., K.Y.H.

## Funding

The authors have nothing to report.

## Conflicts of Interest

The authors declare no conflicts of interest.

## Data Availability

The data that support the findings of this study are available from the corresponding author upon reasonable request.
